# Is rapid scientific publication also high quality? Bibliometric analysis of highly disseminated COVID‐19 research papers

**DOI:** 10.1002/leap.1403

**Published:** 2021-06-01

**Authors:** Amandeep Khatter, Michael Naughton, Hajira Dambha‐Miller, Patrick Redmond

**Affiliations:** ^1^ School of Population Health and Environmental Sciences King's College London London UK; ^2^ School of Primary Care Population Sciences and Medical Education (PPM) University of Southampton UK; ^3^ Department of General Practice Royal College of Surgeons in Ireland Dublin Ireland

**Keywords:** altmetrics, coronavirus Infections, COVID‐19, pandemics, publication impact, quality

## Abstract

The impact of COVID‐19 has underlined the need for reliable information to guide clinical practice and policy. This urgency has to be balanced against disruption to journal handling capacity and the continued need to ensure scientific rigour. We examined the reporting quality of highly disseminated COVID‐19 research papers using a bibliometric analysis examining reporting quality and risk of bias (RoB) amongst 250 top scoring Altmetric Attention Score (AAS) COVID‐19 research papers between January and April 2020. Method‐specific RoB tools were used to assess quality. After exclusions, 84 studies from 44 journals were included. Forty‐three (51%) were case series/studies, and only one was an randomized controlled trial. Most authors were from institutions based in China (*n* = 44, 52%). The median AAS and impact factor was 2015 (interquartile range [IQR] 1,105–4,051.5) and 12.8 (IQR 5–44.2) respectively. Nine studies (11%) utilized a formal reporting framework, 62 (74%) included a funding statement, and 41 (49%) were at high RoB. This review of the most widely disseminated COVID‐19 studies highlights a preponderance of low‐quality case series with few research papers adhering to good standards of reporting. It emphasizes the need for cautious interpretation of research and the increasingly vital responsibility that journals have in ensuring high‐quality publications.

**Key points**

- An examination of highly visible COVID‐19 research articles reveals that 55% could be considered at risk of bias.
- Only 11% of the evaluated early studies on COVID‐19 adhered to good standards of reporting such as PRISMA or CONSORT.
- There was no correlation between quality of reporting and either the journal Impact Factor or the article Altmetric Attention Score in early studies on COVID‐19.
- Most highly visible early articles on COVID‐19 were published in the *Lancet* and *Journal of the American Medical Association*.

## INTRODUCTION

The identification of the SARS‐CoV‐2 virus in December 2019 and the subsequent pandemic has led to a huge research effort in areas of diagnosis, disease prevention, behavioural science, prediction, and treatment. This has resulted in an unprecedented surge in the pace of scientific publishing (Gazendam et al., 2020; Kambhampati et al., 2020). By June 2020, over nineteen thousand research papers had been published on COVID‐19 (Zyoud & Al‐Jabi, 2020). The World Health Organization (WHO) in February 2020 warned of a COVID‐19 ‘infodemic’ with an ‘over‐abundance of information—some accurate and some not’ (WHO, 2020b). Subsequent action by governments worldwide on issues such as social restrictions and wearing of facemasks has led to much debate on the certainty of evidence guiding policy development and clinical practice.

A journal's impact factor or number of citations is traditionally used as a measure of the extent of the research dissemination (Garfield, 2006). However, the delay between publication and the availability of citation metrics has led to the increasing use of alternative measures. The Altmetric score, for example, aims to more fully quantify the non‐traditional means of article dissemination to include websites, blogs, traditional media or social media alongside reference management software such as Mendeley (Brigham, 2014). This purports to represent a societal measure of ‘public engagement’ with research output and provides an indication of research visibility (Bornmann, 2014; Elmore, 2018a; Finch et al., 2017). There is some evidence of correlation between Altmetric scores, future citations and impact factor scores (Costas et al., 2015). Costas et al. found that Altmetric scores can identify extensively cited articles that are also mentioned in non‐journal sources with good precision, especially those that have been recently published. During the pandemic however, the influence of social media and its potential for rapid dissemination and sometimes disinformation has been highlighted (Haneef et al., 2015; WHO, 2020b).

Concerns about the quality and trustworthiness of published research have long been expressed with up to 85% of health research suggested to be ‘wasted’ due to poor design, reporting *etc*. (Chalmers & Glasziou, 2009). COVID‐19 research in particular has faced many of the challenges of early evidence with studies that have poor design selection, unrecognized biases, repeated and multiple analyses, financial incentives, and so called data dredging (Glasziou et al., 2020; Ioannidis, 2005; Young et al., 2008). Scientific journals have a responsibility to balance the selection of well conducted studies with the speed required to keep up with a rapidly evolving global pandemic. Many high profile journals (e.g., *The Lancet*, *BMJ*) have responded by making content freely available with publishing fees waived, providing dedicated calls, encouraging preprint dissemination and guaranteeing rapid editorial decisions (Brown & Horton, 2020). These developments have led to some precipitous publications which have been criticized. Examples include the unfulfilled benefits of hydroxychloroquine treatment, smoking's protective effect on COVID‐19, and the questioning of the veracity of a series of papers related to Surgisphere (Ledford, 2020; The Lancet Editors, 2020; van Schalkwyk et al., 2020). This has spurred interest in the monitoring of this trend with websites such as Retraction Watch gaining a recent large following (Retractionwatch.com, 2020).

## OBJECTIVE

The aim of this study was to characterize highly disseminated (as determined by Altmetric score) COVID‐19 scientific research papers published within scientific journals between 1 January 2020 and 28 April 2020 and to appraise their reporting quality.

## METHODS

### Study design

A bibliometric analysis of highly disseminated COVID‐19 research papers published in scientific journals.

### Eligibility criteria

We included COVID‐19 research studies published between 1 January and 28 April 2020 in scientific journals. All study designs were included. We excluded non‐English language papers, non‐human studies, basic science studies, non‐research papers (editorials, letters, new reports) and preprints.

### Search strategy

On 11 June 2020, we searched the Altmetric Explorer database of indexed articles using medical subject headings (MeSH) alongside the keywords ‘COVID‐19’ and ‘coronavirus’ between 1 January 2020 and 28 April 2020. There were 5,971 attention highlights for articles containing COVID‐19 and 2,523 attention highlights for articles containing coronavirus. The list of identified research papers was deduplicated (891 removed, leaving 7,603) and listed in descending order of Altmetric Attention Score (AAS) with the top 250 highest scoring results retained.

### Selection of studies

A combination of two authors independently screened titles and abstracts using Covidence to ensure consistency with inclusion criteria. Covidence is a web‐based software platform that streamlines the production of systematic reviews. Any papers not meeting the inclusion criteria were excluded at this stage (with reasons for exclusion documented). If there was any uncertainty, consensus was reached by discussion or arbitration by a third reviewer.

Cohen's kappa coefficient (κ) was used to assess inter‐rater agreement. Agreement values were interpreted as follows: above 0.80 = very good agreement, 0.60–0.80 = reasonable agreement, 0.40–0.60 = moderate agreement, and <0.40 = fair to poor agreement (Gwet, 2014).

Following this, a combination of two reviewers independently assessed the full text research papers to ensure studies still fulfilled the inclusion criteria.

### Data extraction and management

A combination of two reviewers undertook data extraction using a predefined data collection checklist adapted from Covidence. Where possible, data on the following was extracted—main author, journal name, journal impact factor (Journal Citation Reports 2020 [Clarivate Web of Science, 2020]), AAS of the paper, study design, country of origin, funding and ethics statement, and use of formal reporting framework (Sample checklist available in Data S1).

### Assessment of study quality

Quality appraisal was determined by the use of a critical appraisal tool appropriate to the study design (e.g., Joanna Briggs Institute critical appraisal tools; PROBAST) (The Joanna Briggs Institute, 2017; Wolff et al., 2019). A combination of two reviewers independently performed the quality assessment. Disagreement was resolved by discussion, and where necessary, by arbitration with a third reviewer. The overall assessment of quality encapsulated the risk of bias (RoB) (as determined by the aforementioned scoring systems) and adherence to reporting frameworks (e.g., PRISMA, CONSORT, STROBE) where appropriate (Moher et al., 2009; Schulz et al., 2010; von Elm et al., 2007).

### Statistical methods

We summarized included study characteristics using basic descriptive statistics. Using the Kruskal‐Wallis test, we evaluated the association between the RoB and both the impact factor of the journal and AAS. All calculations were done in Stata/MP 14.0 (Stata, College Station, TX). Data are presented as medians and interquartile ranges. A significance level of α = 0.05 was used for all the comparisons.

## RESULTS

### Search results

**FIGURE 1:**
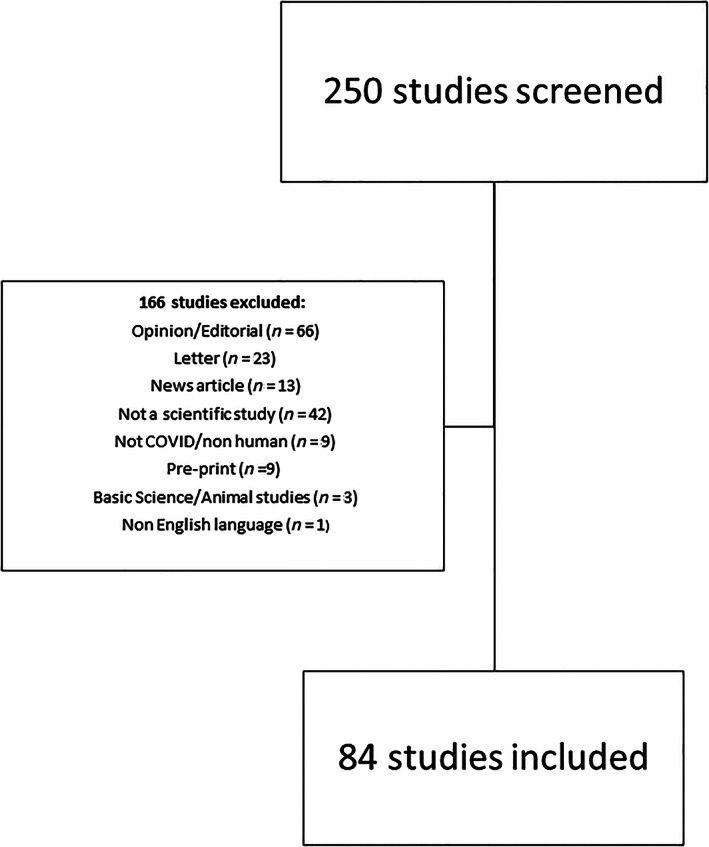
Flow chart of included studies.

A total of 7,602 research papers were identified by searches. The top 250 highest AAS scoring papers were retained (AAS ≥ 733). One hundred and sixty‐six papers were excluded following full text screening, most commonly because they were either opinion pieces, editorials or letters (*n* = 89, 36%) (Fig. 1). Inter‐rater agreement ranged from moderate to very good (0.58–0.94).

### Description of included studies

#### Impact factor and AAS

Eighty‐four studies were included across 44 different scientific journals (Table 1). *The Lancet* (*n* = 9, 11%) and the *Journal of the American Medical Association* (*JAMA*) (*n* = 8, 10%) were the most represented journals with 27 other journals reporting only one study each. The median journal impact factor was 12.8 (interquartile range [IQR] 5.0–44.2) and studies had a median AAS of 2015 (IQR 1,105–4,051.5).

**TABLE 1:** Details of included studies.

	*N* (%^a^)
Total no. of included studies	84
Study design	
Case series	34 (40)
Modelling study	18 (21)
Case studies	9 (11)
Retrospective cohort studies	7 (8)
Reviews	7 (8)
Quasi‐experimental studies	4 (5)
Surveys	2 (2)
RCTs	1 (1)
Diagnostic accuracy studies	1 (1)
Quantitative descriptive	1 (1)
Country of origin	
China	44 (52)
Multinational	11 (13)
United States	8 (10)
United Kingdom	5 (4)
France	3 (4)
Italy	3 (4)
Japan	3 (4)
Others	7 (7)
Journal	
*The Lancet*	9 (11)
*JAMA*	8 (10)
*JAMA Open*	4 (5)
*NEJM*	4 (5)
*Science*	4 (5)
Others	55 (64)
Funding statement	
Yes	71 (85)
Reporting framework acknowledged	
Yes	9 (11)
Ethics declaration	
Yes	72 (86)

Abbreviation: RCT, randomized controlled trial.

^a^ Nearest whole number.

#### Study design

Thirty‐four case series (40%), eighteen modelling studies (21%), nine case studies (11%) and one randomized controlled trial (RCT) were included. The case series provided reports of patients with COVID‐19, usually describing the course of the condition, complications or management, for example, complement associated microvascular injury and thrombosis in the pathogenesis of severe COVID‐19 infection: a report of five cases (Magro et al., 2020). The modelling studies included epidemiological models which tracked COVID‐19 transmission or fatality and prediction models which analysed diagnosis, prognosis or risk, for example, estimated effectiveness of symptom and risk screening to prevent the spread of COVID‐10 (Gostic et al., 2020).

#### Country of origin

The majority of studies' authors originated in China (*n* = 44, 52%), with most modelling studies (78%) having an international authorship.

#### Ethics and funding statements

Funding statements were provided in 71 (85%) of the papers with 72 (86%) including reference to or no requirement for ethical approval.

#### Quality assessment of included studies.

**FIGURE 2:**
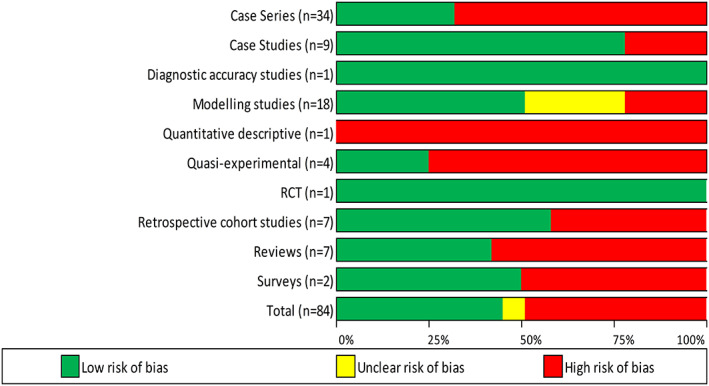
Risk of bias of included studies. RCT, randomized controlled trial.

The majority of studies (*n* = 46, 55%) were deemed to be at high or unclear RoB (Fig. 2). Twenty‐three (68%) of the 34 cases series were deemed at high RoB. Conversely, the single RCT included was at low RoB. The areas contributing most to this high RoB in the case series were the inclusion criteria (50%), the complete (79%) and consecutive (71%) inclusion of participants and the outcomes/follow up (50%) of the participants (Data S1). With regards to the case studies, 89% poorly described the demographics of the participant. Four of the seven review studies lacked high quality critical appraisal, assessment of publication bias and methods to minimize errors in data extraction. Half of the modelling studies had inadequate analysis of their models. All of the retrospective cohort studies failed to address confounding factors.

Nine of the studies (11%) acknowledged the use of a reporting framework and 44% of these had poor or inadequate adherence to their respective framework (Data S1) (Borba et al., 2020; Cai et al., 2020; Castagnoli et al., 2020; Lai et al., 2020; Luo et al., 2020; MacIntyre & Chughtai, 2020; Nussbaumer‐Streit et al., 2020; Sajadi et al., 2020; Wynants et al., 2020). Two of the studies used CONSORT and neither described the limitations or generalisability of their paper (Borba et al., 2020; Cai et al., 2020; Schulz et al., 2010). Four reviews used PRISMA, of which 75% did not include RoB across included studies nor details of a review protocol (Castagnoli et al., 2020; MacIntyre & Chughtai, 2020; Moher et al., 2009; Nussbaumer‐Streit et al., 2020; Wynants et al., 2020).

#### Impact factor and association with RoB

We found no association between either journal impact factor (*p* = 0.912) or AAS (*p* = 0.892) and the RoB judgement.

## DISCUSSION

### Summary

This bibliometric study showed that in the first 4 months of the COVID‐19 pandemic, the most widely disseminated studies were predominantly poor‐quality case series that did not adhere to formal reporting guidelines even though they were published in some of the highest impact medical journals. We found no relationship between impact factor and the RoB.

### Interpretation and context

Accuracy and trustworthiness of published findings are important, especially during a global pandemic. The urgent need for evidence to inform decision making and the (mis)interpretation of research papers in the lay media have been features of this pandemic. This dissemination has taken many forms with a deluge of preprints, press releases, and social media commentary (Saitz & Schwitzer, 2020), compounding this is the capacity of journals to respond at pace which has been severely tested. For example, *JAMA* reported a tripling of submissions, all most all COVID related, in the first 6 months of 2020 (Bauchner et al., 2020).

A number of studies have used bibliometric methods to examine COVID‐19 research. Describing the country (Chahrour et al., 2020; Lou et al., 2020; Mao et al., 2020; Zhai et al., 2020), evidence type (Di Girolamo & Meursinge Reynders, 2020; Gazendam et al., 2020; Li et al., 2020; Odone et al., 2020; Zyoud & Al‐Jabi, 2020), and journal (Chen et al., 2020; DE Felice & Polimeni, 2020; Zhang et al., 2020) specific contribution to research, or contrasting English and Chinese language publications (Fan et al., 2020; Raynaud et al., 2021). In keeping with our findings, they report the bulk of research activity unsurprisingly arising from China in the early stages of the pandemic with an over representation in the top rank biomedical journals. Our study concentrated on publications that were highly disseminated, as determined by AAS. Whilst some of the previous reports categorized the evidence type being presented (e.g., opinion pieces, observational) almost none attempted an in‐depth critical appraisal as we have done. Similar to Raynaud et al., 2021's meta‐research of more than 10,000 COVID‐19 research papers, we found that the majority of identified COVID‐19 papers showed a high degree of bias and importantly, 57.6% did not report primary data (e.g., editorials, news articles) (Raynaud et al., 2021). We have developed this further by describing the key elements contributing to the high RoB in COVID‐19 papers, for example, lack of reporting of demographic information in case series. Additionally, we analysed use of and adherence to reporting frameworks, establishing that only nine studies reported using one and 44% of these had poor or inadequate compliance to their listed framework. Finally, our study found no indication that high impact journals published low RoB COVID‐19 studies.

### Limitations

Our results should be interpreted within the scope of the searches performed and is limited by inclusion of English language papers only. This was not a systematic review and the inclusion strategy was purposely limited given time and resource limitations. The exclusion of preprints is important due to their potential impact on practice (in the absence of peer review) and increasing popularity as a source of information. Our study used only one metric of dissemination (AAS), which includes a composite of many increasingly influential media outlets (e.g., Twitter). In addition, AAS and impact factor scores have been found to have moderate correlation and may predict future citation counts (Costas et al., 2015; Thelwall & Nevill, 2018).

The included research papers covered the early period of the pandemic—it might be expected therefore, that studies requiring more rigorous planning and reporting (e.g., RCTs) would not have been available so early in the emergence of the global pandemic. Indeed, the literature in this area is rapidly evolving with new COVID‐19 relevant publications being released daily. Nevertheless, other reviews have similarly reported only limited numbers of small participant RCTs contributing to the topic (Raynaud et al., 2021).

Finally, a pragmatic decision was made to adapt existing widely used and easily interpretable critical appraisal tools to generate a summary RoB for each of the included study designs. It is possible alternate tools may have varied in their estimation of bias. Nevertheless, the screening and appraisal process was carried out in dual with good inter‐rater reliability.

### Implications

Many prominent biomedical journals have provided rapid publishing, reduced fees and specific COVID‐19 paper calls (Brown & Horton, 2020). Quality of published studies, particularly observational studies, during the pandemic have been reported to be lower quality than matched studies from before the pandemic (Elgendy et al., 2021; Jung et al., 2021). The time to publication has been rapidly shortened with Gazendam et al. reporting a median time from submission to publication of 13 days (Gazendam et al., 2020). Similar findings of decreased publication times were reported during the Ebola epidemic, indicating a common theme of fast publications during public health emergencies (Palayew et al., 2020; Ronit, 2017). Our findings suggest that speed has not necessarily been matched with quality amongst highly disseminated COVID‐19 publications. Indeed, some have called for a flattening of the ‘infodemic curve’ with a greater focus on basic research, systematic reviews, and experimental studies to guide clinical decisions and policy making (Gazendam et al., 2020). For example, the WHO has developed guidance, working with digital companies and social media platforms, to prioritize science‐based health messages (WHO, 2020a).

With so much information available, highlighting good quality experimental evidence that could have substantial clinical impact may be difficult to distinguish. The design, execution and publishing of high quality studies necessarily takes time; and would appear to already be in train with 728 COVID‐19 studies registered (Maguire et al., 2020). Indeed, there have already been successful examples of COVID‐19 practice changing RCTs, for example, vaccines, dexamethasone (Jackson et al., 2020; The RECOVERY Collaborative Group, 2020). The traditional reasoning that, in an emergency, all possible therapies should be tried, irrespective of evidence, has been challenged by the pace of the pandemic with a shift towards rapidly co‐opting of therapies into investigative studies (Lane & Fauci, 2020)—the RECOVERY trial in the United Kingdom being an example of multiple treatment arms (ISRCTNregistry, 2020).

This study's examination of reporting quality has implications for the peer‐review process. We have detailed which specific elements of individual study types are contributing to a higher RoB and which areas of reporting frameworks are commonly not adhered to. Therefore, our results may provide guidance for authors, reviewers, and publishers on improving the quality of published articles. Peer review has a 300 year old history of attempting to filter the ‘irrelevant, trivial, weak, misleading, or potentially harmful content’ (Jefferson, 2002). However, it is both time intensive and expensive, protracted (typically 125 days to publication) and reliant on the generally unpaid and presumed skilled of academics (Powell, 2016). It has evolved with incentivization of peer reviewers, and the introduction of patient reviewers (Schroter et al., 2018), and open publishing of peer review findings (Gasparyan et al., 2015). The acceptability of the traditional peer review process has also come under pressure—not only in identifying reviewers but particularly those with appropriate skills. For example, the *Surgisphere* retractions has led the *Lancet* to insist on data science specialists to review large dataset studies (The Lancet Editors, 2020). Many journals have altered their own processes of review—with *JAMA* requiring internal review only for ‘simple and straightforward’ COVID‐19 studies (Bauchner et al., 2020). An increasing focus is also now on the post publication process encouraging critical commentary and even potential amendments/retractions (Bauchner et al., 2020).

*The Lancet* has claimed that in the absence of peer review ‘the whole edifice of scientific research and publication would have no foundation’ (*The Lancet*, 2008). Preprints are an example of non‐peer reviewed findings which have polarized academics– and pose a challenge to the peer‐review process (Brainard, 2018). Whilst not examined directly, nine out of the one hundred and sixty‐six papers (5%) excluded from our study were preprints. Preprint servers (e.g., medRxiv, bioRxiv) accelerate access as well as the potential misinterpretation of results, and are a prominent part of the scientific community response to urgent health crises (e.g., Ebola, Zika, COVID‐19) (Johansson et al., 2018; Peiperl, 2018). Supporters argue that the transparency and critique received at preprint stage improves the final manuscript (Elmore, 2018b; Fry et al., 2019). Whilst conversely, it is claimed that the misleading nature of some of these early findings may be damaging (Yeo‐Teh & Tang, 2021), as retractions continue to be a rarity and on average take 3 years (Abritis et al., 2021). Retractionwatch.com has led a highly visible campaign to record these retractions, expressions of concerns and corrections (Bramstedt, 2020; Retractionwatch.com, 2020). Concern also exists for the potential misuse of these platforms by vested interests (Ulucanlar et al., 2016) and open publishing itself is further evolving to include initiatives like funder managed platforms, for example, Wellcome Open Research—allowing preprint publishing, bundling peer review and bypassing journal submissions (Kiley, 2020). In light of this and the preponderance of non‐data publications disseminated during the pandemic, future research could build on our results by determining the quality of these articles.

## CONCLUSION

This review of the most widely disseminated COVID‐19 research papers at the early stage of the pandemic shows a preponderance of low‐quality case series with few studies adhering to good standards of reporting. Poor quality research is not new and emphasizes, with greater information availability, the need for adherence to established good practice in transparency of reporting, that is, funding, competing interests, protocol registration. As subsequent waves of the pandemic occur, these findings highlight the need for cautious interpretation of research and emphasize the increasingly vital role and responsibility that journals have in ensuring rigorous high‐quality publications particularly during a pandemic.

## CONFLICT OF INTEREST

Patrick Redmond is an editorial fellow at the BJGP Open with his services provided on a *pro bono* basis. HDM is the editor‐in‐chief of BJGP Open, associate editor and an editorial board member of the British Journal of General Practice.

## AUTHOR CONTRIBUTIONS

**Amandeep Khatter**: Design and implementation of the research; analysis of the results; provided critical feedback; helped shape the final manuscript. **Michael Naughton**: Design and implementation of the research; analysis of the results; provided critical feedback; helped shape the final manuscript. **Hajira Dambha‐Miller**: Conceived the study idea; provided critical feedback; helped shape the final manuscript. **Patrick Redmond**: Conceived the study idea; design and implementation of the research; analysis of the results; lead in writing the manuscript; provided critical feedback; helped shape the final manuscript.

## Supporting information


**Data S1.** Supporting Information.

## Data Availability

The authors confirm that the data supporting the findings of this study are available within the article and its supplementary material.
