# Supplementary material for: Is rapid scientific publication also high quality? Bibliometric analysis of highly disseminated COVID‐19 research papers
Source: Learn Publ. 2021 Jun 1;34(4):568–77. doi: 10.1002/leap.1403 (PMC8242915; doi:10.1002/leap.1403)
Supplement: Supplementary file 1 — Data S1. Supporting Information. [file LEAP-34-568-s001.docx]

**Supplementary materials**

**Is rapid scientific publication also high quality? Bibliometric analysis of highly disseminated COVID-19 research papers.**

Amandeep Khatter^1^, Michael Naughton^1^, Hajira Dambha-Miller ^2^, Patrick Redmond ^1, 3^

^1^ School of Population Health & Environmental Sciences, King’s College London

^2^ Editor in Chief, BJGP Open

^3^ Department of General Practice, Royal College of Surgeons in Ireland, Dublin, Ireland.

**Corresponding author**

Dr Patrick Redmond, NIHR ACL in General Practice, King’s College London

[Patrick.redmond@kcl.ac.uk](mailto:Patrick.redmond@kcl.ac.uk)

**Supplementary Material**

*Data extraction sheet*

| Study ID |  |
| --- | --- |
|  | Lead investigator |
|  | Institution |
|  | Country |
|  | Sponsorship |
|  | Ethics |
|  | Journal |
|  | Reporting Guideline |
|  | Research method |

*Critical Appraisal Tools*

| Study Design | Checklist used |
| --- | --- |
| Case Series | JBI Checklist for Case Series |
| Case Study | JBI Checklist for Case Reports |
| Modelling study | PROBAST: A Tool to Assess the Risk of Bias and Applicability of Prediction Model Studies |
| Retrospective cohort study | JBI Checklist for Cohort Studies |
| Review/Systematic review | JBI Checklist for Systematic Reviews |
| Quasi-experimental study | JBI Checklist for Quasi-Experimental Studies (non-randomized experimental studies) |
| Survey | JBI Checklist for Analytical Cross-Sectional Studies |
| RCT | JBI Checklist for Randomized Controlled Trials |
| Diagnostic accuracy | JBI Checklist for Diagnostic Test Accuracy Studies |
| Quantitative descriptive | JBI Checklist for Case Series |

| Case Series | Inclusion Criteria | Measurement of Condition | Identification of Condition | Consecutive Inclusion of Participants | Complete Inclusion of Participants | Demographics of Participants | Clinical Information of Participants | Outcomes / Follow Up | Site / Clinic Demographic Information | Statistical Analysis | Overall Appraisal |
| --- | --- | --- | --- | --- | --- | --- | --- | --- | --- | --- | --- |
| Ong 2020 | high | low | low | high | high | high | high | high | high | low | high |
| Mao 2020 | low | low | low | low | high | low | low | high | low | low | low |
| Magro 2020 | high | low | low | high | high | low | low | high | high | low | high |
| Luo 2020 | high | low | low | high | high | high | high | high | high | low | high |
| Lu 2020 | high | low | low | high | high | high | low | high | high | low | high |
| Li 2020 | low | low | low | high | high | low | high | high | low | low | high |
| Lei 2020 | high | low | low | high | high | low | low | low | low | low | high |
| Lan 2020 | high | low | low | high | high | high | high | low | high | low | high |
| Inui 2020 | high | low | low | high | high | high | high | high | high | low | high |
| Huang 2020 | low | low | low | low | low | low | low | low | low | low | low |
| Hu 2020 | high | low | low | high | high | low | low | low | low | low | high |
| He 2020 | high | low | low | high | high | high | high | high | high | low | high |
| Guo 2020 | low | low | low | low | high | high | low | high | high | low | high |
| Fang 2020 | low | low | low | low | high | high | high | high | low | low | high |
| Dong 2020 | low | high | low | high | high | low | high | high | low | low | high |
| Danis 2020 | low | low | high | low | low | high | high | high | low | low | high |
| Chen 2020 | high | low | low | high | high | low | low | low | low | low | low |
| Chen 2020 | low | low | low | low | low | low | low | low | low | low | low |
| Zhu 2020 | high | low | low | high | high | high | high | low | low | low | high |
| Zhou 2020 | high | low | low | high | high | high | high | high | high | low | high |
| Zeng 2020 | low | low | low | low | low | low | low | high | high | low | low |
| Zeng 2020 | low | low | low | high | high | high | high | low | low | low | high |
| Yu 2020 | high | low | low | high | high | low | high | high | low | low | high |
| Wei 2020 | low | low | low | low | low | low | low | high | high | low | low |
| Wang 2020 | low | low | low | low | low | low | low | low | low | low | low |
| Su 2020 | high | low | low | high | high | low | low | high | high | low | high |
| Shi 2020 | high | low | low | high | high | low | low | low | high | low | high |
| Shen 2020 | low | low | low | high | high | low | low | low | low | low | low |
| Chen 2020 | low | low | low | high | high | low | low | low | low | low | high |
| Chen 2020 | low | low | low | high | high | low | low | low | low | low | low |
| Chang 2020 | low | low | low | high | high | high | high | low | high | low | high |
| Chan 2020 | high | low | low | high | high | low | low | low | low | low | low |
| Cai 2020 | low | low | low | low | low | low | low | low | low | low | low |
| Barton 2020 | high | low | low | high | high | low | low | low | low | low | high |

djdj

*Risk of Bias in Case Series*

*Risk of Bias in Case Studies*

| Case Study | Demographics of Participants | Clinical History of Participants | Clinical Condition of Participants | Diagnostic Tests / Assessment Methods | Intervention / Treatment | Post - Intervention Clinical Condition | Adverse Events Identified | Takeaway Lessons | Overall Appraisal |
| --- | --- | --- | --- | --- | --- | --- | --- | --- | --- |
| Poyiadji 2020 | high | high | low | low | low | high | high | low | high |
| Kam 2020 | high | low | low | low | low | low | low | low | low |
| Jones 2020 | high | low | low | low | low | low | low | low | low |
| Inciardi 2020 | high | low | low | low | low | low | low | low | low |
| Holshue 2020 | low | low | low | low | low | low | low | low | low |
| Ghinai 2020 | high | high | low | low | low | low | low | low | high |
| Xu 2020 | high | low | low | low | low | low | low | low | low |
| Wu 2020 | high | low | low | low | low | low | low | low | low |
| Colavita 2020 | high | low | low | low | low | low | low | low | high |

*Risk of Bias in Diagnostic Accuracy Study*

*Risk of Bias in Reviews*

| Review | Review Question | Inclusion Criteria | Search Strategy | Sources and Resources | Appraisal Criteria | Appraisal Method | Error-Minimisation Method | Method to Combine Studies | Publication Bias Assessment | Recommendations for Policy and/or Practice | Directives for New Research | Overall Appraisal |
| --- | --- | --- | --- | --- | --- | --- | --- | --- | --- | --- | --- | --- |
| Nussbaumer-Streit 2020 | low | low | low | low | low | low | low | N/A | high | low | low | low |
| MacIntyre 2020 | low | low | low | low | high | high | high | N/A | low | low | high | high |
| Liu 2020 | unclear | high | unclear | unclear | high | high | high | N/A | high | N/A | high | high |
| Kampf 2020 | unclear | high | low | high | high | high | high | N/A | high | low | high | high |
| Wynants 2020 | low | low | low | low | low | low | low | N/A | low | low | low | low |
| Castagnoli 2020 | low | low | low | low | low | low | low | N/A | unclear | low | low | low |
| Sanders 2020 | unclear | low | unclear | low | high | high | high | N/A | high | low | low | high |

| Diagnostic Accuracy Study | Sample of Patients | Avoided Case Control Study | Avoided Inappropriate Exclusions | Index Test Results | Pre-Specified Threshold | Reference Standard | Reference Standard Results | Interval between Index Test and Reference Standard | Participants Received Same Reference Standard | Inclusion of Participants | Overall Appraisal |
| --- | --- | --- | --- | --- | --- | --- | --- | --- | --- | --- | --- |
| Ai 2020 | low | high | low | low | low | low | low | low | low | low | low |

*Risk of Bias in Modelling Studies*

| Modelling Study | Prediction Model Type | Selection of Participants | Concern that the Included Participants and Setting do not Match the Review Question | Predictors or their Assessment | Concern that the Definition, Assessment or Timing of Predictors do not Match the Review Question | Concern that the Outcome does not Match the Review Question | Analysis | Outcome or its Determination | Overall Appraisal |
| --- | --- | --- | --- | --- | --- | --- | --- | --- | --- |
| Quilty 2020 | development | high | high | unclear | low | low | high | unclear | high |
| Pathak 2020 | development | low | low | unclear | low | low | high | high | unclear |
| Nishiura 2020 | development | high | low | unclear | low | low | low | high | unclear |
| Mizumoto 2020 | development | low | low | unclear | unclear | low | high | high | unclear |
| Li 2020 | development | low | low | low | low | low | low | low | low |
| Leung 2020 | development | low | low | unclear | low | low | low | low | low |
| Kraemer 2020 | development | low | low | unclear | low | low | unclear | low | low |
| Hellewell 2020 | development | unclear | high | high | low | low | high | low | unclear |
| Hanlon 2020 | development | low | high | high | high | low | high | unclear | high |
| Gostic 2020 | development | low | low | unclear | low | low | high | unclear | low |
| Gilbert 2020 | development | low | low | unclear | high | low | low | low | low |
| Endo 2020 | development | high | low | high | low | low | low | high | unclear |
| Chinazzi 2020 | development | low | low | unclear | low | low | unclear | unclear | low |
| Zhang 2020 | development | high | high | unclear | low | low | high | low | high |
| Wu 2020 | development | high | high | unclear | unclear | low | high | unclear | high |
| Tian 2020 | development | low | low | unclear | low | low | low | low | low |
| Russell 2020 | development | low | low | unclear | low | low | low | low | low |
| Rocklov 2020 | development | low | low | high | low | low | high | low | low |

*Risk of Bias in Randomised Controlled Trial*

*Risk of Bias in Quasi Experimental Studies*

| Quasi Experimental Study | Cause and Effect | Similar Participants included in any Comparisons | Were the Similar Treatment / Care of Participants Included in any Comparisons | Control group | Multiple measurements of the outcome both pre and post the intervention/exposure | Complete Follow Up | Measurements of Outcomes of Participants Included in any Comparisons | Reliable Measurement of Outcomes | Statistical Analysis | Overall Appraisal |
| --- | --- | --- | --- | --- | --- | --- | --- | --- | --- | --- |
| Cai 2020 | low | low | low | high | high | low | low | unclear | low | low |
| Duan 2020 | high | unclear | high | low | high | low | unclear | unclear | unclear | high |
| Grein 2020 | low | unclear | high | high | low | high | high | low | high | high |
| Gautret 2020 | high | high | unclear | low | high | high | low | high | unclear | high |

| Randomised Controlled Trial | True Randomisation | Concealment of Allocation to Groups | Similar Treatment Groups at Baseline | Participants Blind to Treatment Assignment | Those Delivering Treatment Blind to Treatment Assignment | Outcomes Assessors Blind to Treatment Assignment | Treatment Groups Treated Identically other than the Intervention of Interest | Complete Follow Up | Analysis of Participants in the Groups to which they were Randomised | Similar Measurement of Outcomes between Groups | Reliable Measurement of Outcomes | Statistical Analysis | Appropriate Trial Design | Overall Appraisal |
| --- | --- | --- | --- | --- | --- | --- | --- | --- | --- | --- | --- | --- | --- | --- |
| Borba 2020 | unclear | low | low | low | low | low | low | low | low | low | unclear | low | high | low |

*Risk of Bias in Quantitative Descriptive Study*

| Quantitative Descriptive Study | Inclusion Criteria | Measurement of Condition | Identification of Condition | Consecutive Inclusion of Participants | Complete Inclusion of Participants | Demographics of Participants | Clinical Information of Participants | Outcomes / Follow Up | Site / Clinic Demographic Information | Statistical Analysis | Overall Appraisal |
| --- | --- | --- | --- | --- | --- | --- | --- | --- | --- | --- | --- |
| Lee 2020 | low | unclear | unclear | low | high | high | low | low | high | high | high |

| Retrospective Cohort Study | Similar Groups | Similar Measurement of Exposures | Reliable Measurement of Exposures | Confounding Factors | Strategies to Deal with Confounding Factors | Participants Free of Outcomes from the Start | Reliable Measurement of Outcomes | Follow Up Time | Complete Follow Up | Strategies to Address Incomplete Follow Up | Statistical Analysis | Overall Appraisal |
| --- | --- | --- | --- | --- | --- | --- | --- | --- | --- | --- | --- | --- |
| Zhou 2020 | low | low | low | high | high | N/A | low | low | low | N/A | low | low |
| Wu 2020 | low | low | low | high | high | N/A | low | low | low | N/A | low | low |
| Tang 2020 | low | low | low | high | high | N/A | low | low | low | N/A | low | low |
| Simonnet 2020 | low | low | low | high | high | N/A | unclear | high | low | N/A | low | high |
| Shi 2020 | low | low | low | high | high | N/A | unclear | high | low | N/A | low | high |
| Bi 2020 | unclear | low | high | high | high | N/A | low | low | low | N/A | low | low |
| Sajadi 2020 | high | high | high | high | unclear | unclear | unclear | high | low | N/A | unclear | high |

jh

*Risk of Bias in Surveys*

*Risk of Bias in Retrospective Cohort Studies*

| Survey | Inclusion Criteria | Description of Study Subjects and Setting | Measurement of Exposure | Measurement of Condition | Confounding Factors | Strategies to deal with Confounding Factors | Measurement of Outcomes | Statistical Analysis | Overall Appraisal |
| --- | --- | --- | --- | --- | --- | --- | --- | --- | --- |
| Lai 2020 | low | low | unclear | low | low | low | low | low | low |
| Xie 2020 | unclear | low | unclear | low | high | high | low | unclear | high |

*Adherence to the CONSORT*

| **CONSORT**  **Section/Topic** | **Item No** | **Checklist Item** | **Author** | |
| --- | --- | --- | --- | --- |
|  |  |  | **Borba 2010** | **Cai 2020** |
| **Title and abstract** | 1 | (*a*) Identification as a randomised trial in the title | **Yes** | No |
|  |  | (*b*) Structured summary of trial design, methods, results, and conclusions | Yes | No |
| **Introduction** | | | |  |
| Background and Objectives | 2 | (*a*) Scientific background and explanation of rationale | Partial | No |
|  |  | (b) Specific objectives or hypotheses | Partial | No |
| **Methods** | | | |  |
| Trial Design | 3 | (*a*) Description of trial design (such as parallel, factorial) including allocation ratio | Yes | No |
|  |  | (b) Important changes to methods after trial commencement (such as eligibility criteria), with reasons | Yes | N/A |
| Participants | 4 | (*a*) Eligibility criteria for participants | Yes | Yes |
|  |  | (b) Settings and locations where the data were collected | Yes | Yes |
| Interventions | 5 | The interventions for each group with sufficient details to allow replication, including how and when they were actually administered | Yes | Yes |
| Outcomes | 6 | (a) Completely defined pre-specified primary and secondary outcome measures, including how and when they were assessed | Partial | No |
|  |  | (b) Any changes to trial outcomes after the trial commenced, with reasons | Yes | N/A |
| Sample Size | 7 | (a) How sample size was determined | Yes | No |
|  |  | (b) When applicable, explanation of any interim analyses and stopping guidelines | Yes | No |
| Randomisation | 8 | (*a*) Method used to generate the random allocation sequence | Yes | N/A |
|  |  | (*b*) Type of randomisation; details of any restriction (such as blocking and block size) | Yes | N/A |
|  | 9 | Mechanism used to implement the random allocation sequence (such as sequentially numbered containers), describing any steps taken to conceal the sequence until interventions were assigned | Yes | No |
|  | 10 | Who generated the random allocation sequence, who enrolled participants, and who assigned participants to interventions | Yes | N/A |
| Blinding | 11 | (a) If done, who was blinded after assignment to interventions (for example, participants, care providers, those assessing outcomes) and how | Partial | N/A |
|  |  | (b) If relevant, description of the similarity of interventions | Yes | No |
| Statistical Methods | 12 | (a) Statistical methods used to compare groups for primary and secondary outcomes | Yes | Yes |
|  |  | (b) Methods for additional analyses, such as subgroup analyses and adjusted analyses | Partial | Yes |
| **Results** | | | |  |
| Participant Flow | 13 | (a) For each group, the numbers of participants who were randomly assigned, received intended treatment, and were analysed for the primary outcome | Yes | Yes |
|  |  | (b) For each group, losses, and exclusions after randomisation, together with reasons | No | No |
| Recruitment | 14 | (a) Dates defining the periods of recruitment and follow-up | Yes | Yes |
|  |  | (b) Why the trial ended or was stopped | Yes | Yes |
| Baseline Data | 15 | A table showing baseline demographic and clinical characteristics for each group | Yes | Yes |
| Numbers Analysed | 16 | For each group, number of participants (denominator) included in each analysis and whether the analysis was by original assigned groups | Yes | Yes |
| Outcomes and Estimation | 17 | (a) For each primary and secondary outcome, results for each group, and the estimated effect size and its precision (such as 95% confidence interval) | Partial | Partial |
|  |  | (b) For binary outcomes, presentation of both absolute and relative effect sizes is recommended | No | No |
| Ancillary Analyses | 18 | Results of any other analyses performed, including subgroup analyses and adjusted analyses, distinguishing pre-specified from exploratory | No | No |
| Harms | 19 | All important harms or unintended effects in each group | Yes | Yes |
| **Discussion** | | | |  |
| Limitations | 20 | Trial limitations, addressing sources of potential bias, imprecision, and, if relevant, multiplicity of analyses | No | No |
| Generalisability | 21 | Generalisability (external validity, applicability) of the trial findings | No | No |
| Interpretation | 22 | Interpretation consistent with results, balancing benefits, and harms, and considering other relevant evidence | Yes | Yes |
| **Other information** | | | |  |
| Registration | 23 | Registration number and name of trial registry | Yes | Yes |
| Protocol | 24 | Where the full trial protocol can be accessed, if available | Yes | Yes |
| Funding | 25 | Sources of funding and other support (such as supply of drugs), role of funders | Yes | Yes |
| **OVERALL** |  | | Poor | Inadequate |

*Adherence to the Checklist for Reporting Case Series*

| **Checklist for Reporting Case Series**  **Section/Topic** | **Item No** | **Author** |
| --- | --- | --- |
|  |  | **Luo 2020** |
| Explicitly state the hypothesis/hypotheses under consideration | 1 | Yes |
| Explicitly provide eligibility criteria for subjects in the report | 2 | No |
| Precisely describe how treatments were administered or potential risk factors defined | 3 | Yes |
| Compare observed results with those in an appropriate external comparison group; discuss potential biases arising from such comparison | 4 | N/A |
| Perform appropriate statistics, ensuring that assumptions of the statistical methods are reasonable in this setting | 5 | Yes |
| Discuss the biological plausibility of the hypothesis in light of the report's observations | 6 | Yes |
| Explicitly discuss the report's limitations and how these limitations could be overcome in future studies | 7 | Yes |
| **OVERALL** | | Good |

*Adherence to the PRISMA*

| **PRISMA Section / Topic** | **Item No** | **Checklist Item** | **Author** | | | |
| --- | --- | --- | --- | --- | --- | --- |
|  |  |  | **Wynants 2020** | **Castagnoli 2020** | **Macintyre 2020** | **Nussbaumer 2020** |
| **Title** | | | |  |  |  |
| Title | 1 | Identify the report as a systematic review, meta-analysis, or both. | Yes | Yes | Yes | Partial |
| **Abstract** | | | |  |  |  |
| Abstract | 2 | Provide a structured summary including, as applicable: background; objectives; data sources; study eligibility criteria, participants, and interventions; study appraisal and synthesis methods; results; limitations; conclusions and implications of key findings; systematic review registration number. | Yes | Yes | Yes | Yes |
| **Introduction** | | | |  |  |  |
| Rationale | 3 | Describe the rationale for the review in the context of what is already known. | Yes | Yes | Yes | Yes |
| Objectives | 4 | Provide an explicit statement of questions being addressed with reference to participants, interventions, comparisons, outcomes, and study design (PICOS). | Yes | Yes | Partial | Yes |
| **Methods** | | | |  |  |  |
| Protocol and Registration | 5 | Indicate if a review protocol exists, if and where it can be accessed (e.g., Web address), and, if available, provide registration information including registration number. | Yes | No | No | No |
| Eligibility Criteria | 6 | Specify study characteristics (e.g., PICOS, length of follow-up) and report characteristics (e.g., years considered, language, publication status) used as criteria for eligibility, giving rationale. | Yes | Yes | Partial | Yes |
| Information Sources | 7 | Describe all information sources (e.g., databases with dates of coverage, contact with study authors to identify additional studies) in the search and date last searched. | Yes | Yes | Yes | Yes |
| Search | 8 | Present full electronic search strategy for at least one database, including any limits used, such that it could be repeated. | Yes | No | No | Yes |
| Study selection | 9 | State the process for selecting studies (i.e., screening, eligibility, included in systematic review, and, if applicable, included in the meta-analysis). | Yes | Yes | Partial | Yes |
| Data collection process | 10 | Describe method of data extraction from reports (e.g., piloted forms, independently, in duplicate) and any processes for obtaining and confirming data from investigators. | Yes | Yes | No | Yes |
| Data items | 11 | List and define all variables for which data were sought (e.g., PICOS, funding sources) and any assumptions and simplifications made. | Yes | Yes | No | Yes |
| Risk of bias in individual studies | 12 | Describe methods used for assessing risk of bias of individual studies (including specification of whether this was done at the study or outcome level), and how this information is to be used in any data synthesis. | Yes | No | No | Yes |
| Summary measures | 13 | State the principal summary measures (e.g., risk ratio, difference in means). | Yes | No | No | Yes |
| Synthesis of results | 14 | Describe the methods of handling data and combining results of studies, if done, including measures of consistency (e.g., I2) for each meta-analysis. | Yes | N/A | N/A | Yes |
| Risk of bias across studies | 15 | Specify any assessment of risk of bias that may affect the cumulative evidence (e.g., publication bias, selective reporting within studies). | No | No | No | Yes |
| Additional analyses | 16 | Describe methods of additional analyses (e.g., sensitivity or subgroup analyses, meta-regression), if done, indicating which were pre-specified. | N/A | N/A | N/A | N/A |
| **Results** | | | |  |  |  |
| Study selection | 17 | Give numbers of studies screened, assessed for eligibility, and included in the review, with reasons for exclusions at each stage, ideally with a flow diagram. | Yes | Yes | Partial | Yes |
| Study characteristics | 18 | For each study, present characteristics for which data were extracted (e.g., study size, PICOS, follow-up period) and provide the citations. | Yes | Yes | Yes | Yes |
| Risk of bias within studies | 19 | Present data on risk of bias of each study and, if available, any outcome level assessment (see item 12). | Yes | No | No | Yes |
| Results of individual studies | 20 | For all outcomes considered (benefits or harms), present, for each study: (a) simple summary data for each intervention group (b) effect estimates and confidence intervals, ideally with a forest plot. | Yes | N/A | Partial | Yes |
| Synthesis of results | 21 | Present results of each meta-analysis done, including confidence intervals and measures of consistency. | Yes | N/A | Partial | Yes |
| Risk of bias across studies | 22 | Present results of any assessment of risk of bias across studies (see Item 15). | Yes | No | No | Yes |
| Additional analysis | 23 | Give results of additional analyses, if done (e.g., sensitivity or subgroup analyses, meta-regression [see Item 16]). | N/A | N/A | N/A | N/A |
| **Discussion** | | | |  |  |  |
| Summary of evidence | 24 | Summarize the main findings including the strength of evidence for each main outcome; consider their relevance to key groups (e.g., healthcare providers, users, and policy makers). | Yes | Yes | Yes | Yes |
| Limitations | 25 | Discuss limitations at study and outcome level (e.g., risk of bias), and at review-level (e.g., incomplete retrieval of identified research, reporting bias). | Yes | Yes | No | Yes |
| Conclusions | 26 | Provide a general interpretation of the results in the context of other evidence, and implications for future research. | Yes | Yes | No | Yes |
| **Funding** | | | |  |  |  |
| Funding | 27 | Describe sources of funding for the systematic review and other support (e.g., supply of data), role of funders for the systematic review. | Yes | No | Yes | Yes |
| **OVERALL** |  | | Excellent | Poor | Inadequate | Excellent |

*Adherence to the STROBE Statement*

| **STROBE Section/Topic** | **Item No** | **Checklist Item** | **Author** |
| --- | --- | --- | --- |
|  |  |  | **Sajadi 2020** |
| **Title and abstract** | 1 | (*a*) Indicate the study’s design with a commonly used term in the title or the abstract | Yes |
|  |  | (*b*) Provide in the abstract an informative and balanced summary of what was done and what was found | Yes |
| **Introduction** | | | |
| Background/rationale | 2 | Explain the scientific background and rationale for the investigation being reported | Yes |
| Objectives | 3 | State specific objectives, including any prespecified hypotheses | Yes |
| **Methods** | | | |
| Study design | 4 | Present key elements of study design early in the paper | Yes |
| Setting | 5 | Describe the setting, locations, and relevant dates, including periods of recruitment, exposure, follow-up, and data collection | No |
| Participants | 6 | (*a*) Give the eligibility criteria, and the sources and methods of selection of participants. Describe methods of follow-up | Yes |
|  |  | (*b*) For matched studies, give matching criteria and number of exposed and unexposed | N/A |
| Variables | 7 | Clearly define all outcomes, exposures, predictors, potential confounders, and effect modifiers. Give diagnostic criteria, if applicable | Yes |
| Data sources/ measurement | 8* | For each variable of interest, give sources of data and details of methods of assessment (measurement). Describe comparability of assessment methods if there is more than one group | Yes |
| Bias | 9 | Describe any efforts to address potential sources of bias | No |
| Study size | 10 | Explain how the study size was arrived at | No |
| Quantitative variables | 11 | Explain how quantitative variables were handled in the analyses. If applicable, describe which groupings were chosen and why | Yes |
| Statistical methods | 12 | (*a*) Describe all statistical methods, including those used to control for confounding | Yes |
|  |  | (*b*) Describe any methods used to examine subgroups and interactions | Yes |
|  |  | (*c*) Explain how missing data were addressed | N/A |
|  |  | (*d*) If applicable, explain how loss to follow-up was addressed | N/A |
|  |  | (*e*) Describe any sensitivity analyses | Yes |
| **Results** | | | |
| Participants | 13* | (a) Report numbers of individuals at each stage of study—e.g. numbers potentially eligible, examined for eligibility, confirmed eligible, included in the study, completing follow-up, and analysed | Yes |
|  |  | (b) Give reasons for non-participation at each stage | N/A |
|  |  | (c) Consider use of a flow diagram | N/A |
| Descriptive data | 14* | (a) Give characteristics of study participants (e.g. demographic, clinical, social) and information on exposures and potential confounders | Yes |
|  |  | (b) Indicate number of participants with missing data for each variable of interest | N/A |
|  |  | (c) Summarise follow-up time (e.g., average, and total amount) | N/A |
| Outcome data | 15* | Report numbers of outcome events or summary measures over time | Yes |
| Main results | 16 | (*a*) Give unadjusted estimates and, if applicable, confounder-adjusted estimates and their precision (e.g., 95% confidence interval). Make clear which confounders were adjusted for and why they were included | Yes |
|  |  | (*b*) Report category boundaries when continuous variables were categorized | N/A |
|  |  | (*c*) If relevant, consider translating estimates of relative risk into absolute risk for a meaningful time period | N/A |
| Other analyses | 17 | Report other analyses done—e.g. analyses of subgroups and interactions, and sensitivity analyses | N/A |
| **Discussion** | | | |
| Key results | 18 | Summarise key results with reference to study objectives | Yes |
| Limitations | 19 | Discuss limitations of the study, taking into account sources of potential bias or imprecision. Discuss both direction and magnitude of any potential bias | Yes |
| Interpretation | 20 | Give a cautious overall interpretation of results considering objectives, limitations, multiplicity of analyses, results from similar studies, and other relevant evidence | Yes |
| Generalisability | 21 | Discuss the generalisability (external validity) of the study results | Yes |
| **Other information** | | | |
| Funding | 22 | Give the source of funding and the role of the funders for the present study and, if applicable, for the original study on which the present article is based | Yes |
| **OVERALL** |  | | Good |

*Adherence to the American Association for Public Opinion Research Reporting Framework*

| **American Association for Public Opinion Research**  **Section/Topic** | **Item No** | **Author** |
| --- | --- | --- |
|  |  | **Lai 2020** |
| Have specific goals. | 1 | Yes |
| Consider alternative data beyond a survey. | 2 | No |
| Select samples that well represent the population to be studied. | 3 | Yes |
| Use designs that balance costs with errors. | 4 | Yes |
| Take great care in matching question format and wording to the concepts being measured and the population being studied. | 5 | Yes |
| Pre-test questionnaires and procedures. | 6 | No |
| Train interviewers carefully on interviewing techniques and the subject matter of the survey. | 7 | N/A |
| Check quality at each stage. | 8 | Yes |
| Maximize cooperation or response rates within the limits of ethical treatment of human subjects. | 9 | Yes |
| Use appropriate statistical analytic and reporting techniques. | 10 | Yes |
| Develop and fulfil pledges of confidentiality given to respondents. | 11 | Yes |
| Disclose all methods of the survey to allow for evaluation and replication. | 12 | Yes |
| **OVERALL** | | Good |
